# Association Between Circulating Regulator of Calcineurin 2 Concentrations With Overweight and Obesity

**DOI:** 10.3389/fendo.2022.857841

**Published:** 2022-06-06

**Authors:** Hongya Wang, Xia Fang, Qian Ren, Yan Zeng, Xiaozhen Tan, Ting Ye, Jiahao Fan, Yong Xu

**Affiliations:** ^1^ Department of Endocrinology and Metabolism, The Affiliated Hospital of Southwest Medical University, Luzhou, China; ^2^ Cardiovascular and Metabolic Diseases Key Laboratory of Luzhou, Luzhou, China; ^3^ Sichuan Clinical Research Center for Nephropathy, Luzhou, China; ^4^ Metabolic Vascular Disease Key Laboratory of Sichuan Province, Luzhou, China; ^5^ Department of Laboratory Medicine, The Affiliated Hospital of Southwest Medical University, Luzhou, China; ^6^ Department of Gastroenterology, The Affiliated Hospital of Southwest Medical University, Luzhou, China

**Keywords:** RCAN2, overweight, obesity, biomarker, cross-sectional study

## Abstract

**Background:**

Regulator of calcineurin 2 (RCAN2) has been reported to promote food intake and weight gain in animal studies. However, its effect on body weight in humans is unclear.

**Objective:**

This study aimed to investigate the relationship between serum RCAN2 concentrations and participants with overweight/obesity.

**Methods:**

A cross-sectional study was performed in 872 Chinese adults, including 348 participants with normal weight (NW), 397 participants with overweight (OW), and 127 participants with obesity (OB). All participants were divided into NW, OW and OB groups according to their body mass index (BMI). Serum RCAN2 concentrations were determined by enzyme-linked immunosorbent assay.

**Results:**

Serum RCAN2 concentrations gradually increased with the increase of BMI (*p* < 0.001). The percentages of OW/OB gradually increased in tandem with increasing tertiles of RCAN2 (*p* < 0.001). Additionally, serum RCAN2 concentrations were significantly correlated with a series of anthropometric and metabolic parameters, predominantly including body weight, BMI, SBP, DBP, total cholesterol, triglycerides, HDL-C, LDL-C (all *p* < 0.05). Furthermore, logistic regression analysis showed that the risk of OW/OB was significantly increased with the increase of serum RCAN2 concentrations. Receiver operation characteristic (ROC) curve analysis revealed that serum RCAN2, especially serum RCAN2/(AST/ALT) ratio, might serve as a candidate biomarker for obesity.

**Conclusion:**

Serum RCAN2 concentrations were increased in subjects with OW/OB. The increased serum RCAN2 concentrations were associated with the increased risks of OW/OB.

## Introduction

According to the World Health Organization (WHO), overweight (OW) or obesity (OB) refers to the excessive or abnormal accumulation of fat in organs and tissues of the body, thereby increasing health risks (1). Obesity is a risk factor for the development of a large number of diseases, such as type 2 diabetes mellitus (T2DM), non-alcoholic fatty liver disease (NAFLD), cardiovascular disease (CVD), stroke, osteoarthritis, and even cancers, triggering a significant decline in quality of life, life expectancy and disability (2–4). Worldwide, 39% adults with overweight (39% for men and 40% for women), 13% adults with obesity (11% for men and 15% for women) (5). In this context, obesity pandemic has become an important health issue across the globe and may create enormous health and economy burden (6). Effective medications for obesity have been slow to emerge, efforts are still underway to develop new, transformative and potent drugs (7).

Regulator of calcineurin 2 (RCAN2, also known as ZAKI-4, DSCR1L1 or MCIP2) was initially discovered as a thyroid hormone (T_3_)-responsive gene in cultured skin fibroblasts (8), and subsequently reported to inhibit calcineurin-dependent transcriptional responses by binding to the catalytic domain of calcineurin A (9, 10). RCAN2 is expressed ubiquitously in the brain, heart, kidney, liver and skeletal muscle (10, 11). RCAN2 has been found to be involved in the development of many diseases. RCAN2 plays an important role in the development and function of the brain by regulating the function of calcineurin (12). RCAN2 is constitutively expressed in endothelial cells and inhibits VEGF-mediated angiogenesis (13). Additionally, RCAN2 is associated with development, proliferation, and the formation of tumorigenic network of cancers (14–16). Attractively, Sun, et al. found that knockout of RCAN2 gene in the whole organism can ameliorate the age- and diet-induced obesity, glucose tolerance, insulin sensitivity, and hepatic steatosis in mice (17, 18). These results indicate that RCAN2 plays an important role in the development of obesity in mice. So far, no study has explored the relationship between serum RCAN2 concentrations with overweight and obesity in humans.

In this study, we planned to 1) investigate the serum RCAN2 concentrations of 348 participants with normal weight, 397 participants with overweight, and 127 participants with obesity; 2) analyze the correlation between serum RCAN2 concentrations and clinical indicators and metabolic indicators; 3) explore the diagnostic value of serum RCAN2 for discriminating individuals with obesity from controls.

## Study Population and Design

A total of 872 participants were screened from the physical examination center of the affiliated hospital of Southwest Medical University. Subjects were classified into three groups based on their body mass index (BMI): normal weight (NW) group (18.5 ≤ BMI<24 kg/m^2^, overweight (OW) group (24 ≤ BMI ≤ 28 kg/m^2^), and obesity (OB) group (BMI > 28 kg/m^2^) (19). All subjects with any of the following conditions were excluded: secondary causes of obesity, previous history of hyperthyroidism or hypothyroidism, acute and chronic cardiovascular diseases, cancers, general poor health status, stroke and any other serious diseases, pregnancy or breastfeeding, use of any weight-affecting drugs.

All protocols followed the ethical guidelines of the 1964 Declaration of Helsinki and were approved by the Human Research Ethics Committee of the Southwest Medical University Hospital (license number: KY2021086). Informed written consent was obtained from all participants.

### Clinical and Anthropometric Parameters

After overnight fasting, all participants underwent anthropometric measurements with light clothing and no footwear. Body height and weight were measured using an ultrasound analyzer (SK-V7, Shenzhen, China), BMI was calculated as weight (kg)/height (m^2^). Waist circumference (WC) and hip circumference (HC) were measured using a cloth tape. Waist-to-hip ratio (WHR) was calculated as WC (cm)/HC (cm). After resting for at least 5 minutes, systolic blood pressure (SBP), diastolic blood pressure (DBP), and heart rate were measured using a medical automatic electronic blood pressure monitor (HBP-9020, Omron Corporation, Kyoto, Japan). All anthropometric parameters were measured by professionally trained nurses.

### Measurement of Biochemical Parameters

Blood samples were collected from the antecubital vein of all subjects after an overnight fast of 10 to 12 hours and were stored at -80°C until analysis. An automated biochemical analyzer (ADVIA2400, SIEMENS, Germany) was used to detect alanine aminotransferase (ALT), aspartate aminotransferase (AST), total protein (TP), albumin (ALB), globulin (GLO), gamma-glutamyl transpeptidase (GGT), alkaline phosphatase (ALP), urea nitrogen (urea), uric acid (UA), creatinine (Crea), total cholesterol (TC), triglycerides (TG), high-density lipoprotein cholesterol (HDL-C), low-density lipoprotein cholesterol (LDL-C), fasting blood glucose (FBG), and homocysteine (HCY). Peripheral white blood cell (WBC) and neutrophil (NEU) counts were measured using an automated blood cell counter (Myriad BC-6800, Shenzhen, China).

### Measurements of Serum Concentrations of RCAN2

Serum RCAN2 concentrations were quantified using a commercial enzyme-linked immunosorbent assay (ELISA) kit (Human RCAN2 ELISA Kit, Abebio, Wuhan, China) according to the manufacturer’s protocol. Serum samples were diluted 10-fold prior to the assay. The intra- and inter-test variations were less than 8% and less than 12%, respectively. The detection range of the kit is 0.156ng/mL - 10ng/mL.

### Statistical Analyses

Continuous variables are expressed as mean ± standard deviation (SD), Categorial variables were expressed as *n* (%). The differences between groups of continuous variables were evaluated by Student’s *t*-test or Mann-Whitney *U*-test and one-way analysis of variance (ANOVA) or Kruskal-Wallis test. Bonferroni-corrected *p*-values were used in multiple testing. The differences between groups of categorical variables were accessed by Chi-square test. In order to assess the correlation between serum RCAN2 concentrations and other variables, the data was analyzed by Pearson correlation or Spearman correlation test. Multiple liner regression analysis was conducted with naturally logarithmic transformed serum RCAN2 as dependent variable and other parameters as independent variables. Binary logistic regression analysis was performed to explore the risks of serum RCAN2 concentrations for overweight/obesity. The diagnostic value of serum RCAN2 or serum RCAN2/(AST/ALT) ratio for the obesity was accessed by the area under the receiver operating characteristic (ROC) curve (AUC). Two-tailed *p* < 0.05 was considered statistically significant. All statistical analysis and graphics were performed using SPSS 25.0 (SPSS Inc., Chicago, IL, USA) and GraphPad Prism 8.0 (GraphPad Software Inc., San Diego, CA, USA).

## Results

### Baseline Characteristics and Serum RCAN2 Concentrations of All Participants

**Figure 1:**
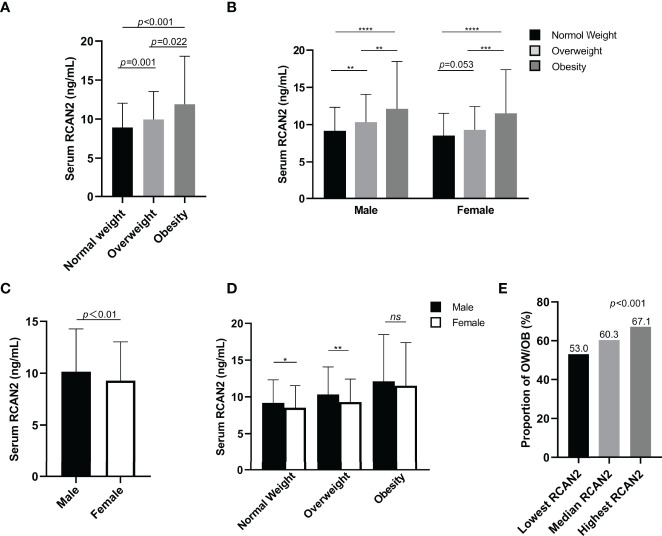
Serum RCAN2 concentrations in subjects with normal weight, overweight and obesity **(A)**. Serum RCAN2 concentrations in males and females with normal weight, overweight and obesity **(B)**. Comparison of the overall level of RCAN2 between men and women **(C)**. Comparison of RCAN2 concentrations in men and women across BMI categories **(D)**. Proportion of overweight or obesity in different concentrations of serum RCAN2 **(E)**. *p* values were derived from Mann–Whitney *U* test for serum RCAN2 concentrations, and chi-square test for the categorical variables. ^*^ *p* < 0.05, ^**^ *p* < 0.01, ^***^ *p* < 0.001, ^****^ *p* < 0.0001, ^ns^ *p* > 0.05.

**Table 1** summarizes the clinical characteristics, biochemical parameters and serum RCAN2 concentrations of all participants. There were significant differences in the age, BW, BMI, WC, HC, WHR, SBP, DBP, WBC, NEU, ALT, AST, AST/ALT, GLO, A/G, TBIL, DBIL, IBIL, GGT, ALP, Urea, UA, TC, TG, HDL-C, LDL-C and FBG (all *p* < 0.05) among the three groups. RCAN2 concentrations were gradually increased (8.94 ± 3.10 ng/mL for NW vs. 9.98 ± 3.57 ng/mL for OW vs. 11.90 ± 6.16 ng/mL for OB, *p* < 0.001) (**Figure 1A**).

**Table 1:** Baseline characteristics of participants according to BMI.

Variables	Normal Weight (*n*=348)	Overweight (*n*=397)	Obesity (*n*=127)	*p* value
**Anthropometric parameters**				
**Male**	218 (62.6)	259 (65.2)	81 (63.8)	–
**Age (year)**	38.63 ± 10.72	40.46 ± 10.62^a^	39.44 ± 10.53	**0.042**
**BW (kg)**	59.61 ± 6.76	69.79 ± 7.31^a^	85.84 ± 16.06^a^ ^b^	**<0.001**
**Height (cm)**	165.00 ± 7.61	164.46 ± 7.69	165.68 ± 9.37	0.397
**BMI (kg/m^2^)**	21.89 ± 1.43	25.75 ± 1.09^a^	31.02 ± 3.39^a^ ^b^	**<0.001**
**WC (cm)**	77.79 ± 5.91	85.30 ± 6.34^a^	96.65 ± 10.23^a^ ^b^	**<0.001**
**Male**	78.69 ± 6.11^c^	87.53 ± 5.38^a^ ^c^	100.04 ± 9.74^a^ ^b^ ^c^	–
**Female**	76.27 ± 5.23	81.13 ± 5.90^a^	90.67 ± 8.20^a^ ^b^	–
**HC (cm)**	93.22 ± 4.86	97.69 ± 4.52^a^	105.94 ± 7.43^a^ ^b^	**<0.001**
**Male**	93.17 ± 4.69	98.06 ± 4.50^a^ ^c^	107.19 ± 7.72^a^ ^b^ ^c^	–
**Female**	93.31 ± 5.16	96.99 ± 4.51^a^	103.76 ± 6.39^a^ ^b^	–
**WHR**	0.83 ± 0.05	0.87 ± 0.05^a^	0.91 ± 0.05^a^ ^b^	**<0.001**
**Male**	0.84 ± 0.05^c^	0.89 ± 0.04^a^ ^c^	0.93 ± 0.05^a^ ^b^ ^c^	–
**Female**	0.82 ± 0.04	0.84 ± 0.51^a^	0.87 ± 0.50^a^ ^b^	–
**SBP (mmHg)**	117.83 ± 14.19	122.94 ± 13.22^a^	130.24 ± 14.68^a^ ^b^	**<0.001**
**DBP (mmHg)**	70.73 ± 9.52	73.82 ± 9.87^a^	79.26 ± 11.18^a^ ^b^	**<0.001**
**Heart Rate**	83.95 ± 11.74	83.17 ± 10.94	85.80 ± 10.56	0.053
**Metabolic parameters**				
**WBC (10^9/L)**	6.00 ± 1.59	6.43 ± 1.44^a^	7.18 ± 1.75^a^ ^b^	**<0.001**
**NEU (10^9/L)**	3.49 ± 1.27	3.74 ± 1.12^a^	4.19 ± 1.26^a^ ^b^	**<0.001**
**ALT (U/L)**	20.99 ± 9.80	31.34 ± 21.47^a^	45.23 ± 42.62^a^ ^b^	**<0.001**
**AST (U/L)**	21.47 ± 5.65	24.69 ± 10.55^a^	28.26 ± 16.08^a^ ^b^	**<0.001**
**AST/ALT**	1.15 ± 0.43	0.93 ± 0.33^a^	0.78 ± 0.29^a^ ^b^	**<0.001**
**TP (g/L)**	72.27 ± 3.43	72.34 ± 3.32	72.78 ± 3.16	0.448
**ALB (g/L)**	46.66 ± 2.33	46.54 ± 2.25	46.3 ± 2.33	0.216
**GLO (g/L)**	25.61 ± 2.62	25.81 ± 2.69	26.48 ± 2.77^a^	**0.016**
**A/G**	1.84 ± 0.21	1.82 ± 0.22	1.77 ± 0.22^a^	**0.008**
**TBIL (μmol/L)**	16.20 ± 6.17	14.95 ± 6.48^a^	14.09 ± 6.24^a^	**<0.001**
**DBIL (μmol/L)**	4.66 ± 1.83	4.15 ± 1.80^a^	3.88 ± 1.66^a^	**<0.001**
**IBIL (μmol/L)**	11.54 ± 4.48	10.80 ± 4.85^a^	10.24 ± 4.78^a^	**<0.001**
**GGT (U/L)**	23.99 ± 19.88	38.31 ± 38.81^a^	49.02 ± 53.28^a^ ^b^	**<0.001**
**ALP (U/L)**	70.00 ± 19.78	73.47 ± 19.78^a^	78.45 ± 19.79^a^ ^b^	**<0.001**
**Urea (mmol/L)**	4.95 ± 1.14	5.09 ± 1.09	4.85 ± 1.02	**0.047**
**UA (μmol/L)**	324.95 ± 77.3	354.42 ± 83.09^a^	383.68 ± 92.92^a^ ^b^	**<0.001**
**Crea (μmol/L)**	66.38 ± 12.48	67.07 ± 12.32	65.46 ± 12.27	0.334
**TC (mmol/L)**	4.75 ± 0.86	4.81 ± 0.87	5.05 ± 0.91^a^ ^b^	**0.007**
**TG (mmol/L)**	1.26 ± 1.00	1.89 ± 1.53^a^	2.50 ± 2.39^a^ ^b^	**<0.001**
**HDL-C (mmol/L)**	1.46 ± 0.35	1.24 ± 0.29^a^	1.14 ± 0.24^a^ ^b^	**<0.001**
**LDL-C (mmol/L)**	3.07 ± 0.88	3.19 ± 0.86	3.49 ± 0.92^a^ ^b^	**<0.001**
**FBG (mmol/L)**	5.05 ± 1.16	5.32 ± 1.14^a^	5.68 ± 1.75^a^	**<0.001**
**HCY (μmol/L)**	11.93 ± 6.76	12.41 ± 7.9	11.95 ± 7.47	0.639
**eGFR(ml/min)**	123.92 ± 21.26	121.92 ± 20.90	126.05 ± 22.99	0.107
**RCAN2 (ng/mL)**	8.94 ± 3.10	9.98 ± 3.57^a^	11.90 ± 6.16^a^ ^b^	**<0.001**
**Male**	8.91 ± 3.03^c^	10.04 ± 3.61^a^ ^c^	11.96 ± 6.13^a^	–
**Female**	8.96 ± 3.07	9.92 ± 3.57	11.90 ± 6.17^a^ ^b^	–

Continuous variables were expressed as mean ± SD. Categorial variables were expressed as n (%). p values were derived from one-way analysis of variance (ANOVA) or Kruskal-Wallis test for continuous variables.

a p< 0.05 compared with normal weight group;

b p < 0.05 compared with overweight group.

c p < 0.05 compared with females in corresponding group. Bold font indicated p < 0.05.

For further analysis, subjects have been divided into 6 groups combined BMI and gender, similar to **Figure 1A**, RCAN2 levels increased progressively with BMI categories in both males and females, although the difference in RCAN2 levels between normal weight and overweight in female subjects approached statistical significance (**Figure 1B**). As shown in **Figure 1C**, men have higher RCAN2 levels in general, compared with women (*p* < 0.01). We also compared the differences in RCAN2 levels between men and women in the same BMI classification, men with normal weight and overweight have significantly higher RCAN2 levels, but this difference was not significant in subjects with obese (**Figure 1D**).

### Clinical Features and Prevalence of OW/OB by Tertiles of Serum RCAN2

All subjects were divided into three groups according to serum RCAN2 tertiles (lowest tertile: < 7.86 ng/mL; median tertile: 7.86-10.63ng/mL; highest tertile: > 10.63ng/mL). As shown in **Supplementary Table 1**, male ratio, age, BW, BMI, WC, WHR, SBP, DBP, ALT, AST, AST/ALT, TP, ALB, DBIL, GGT, UA, TC, TG, HDL-C, LDL-C, FBG, eGFR, and RCAN2 concentrations were significantly different between subjects in different serum RCAN2 tertiles (all *p* < 0.05). As displayed in the **Figure 1E**, the prevalence of OW/OB was rapidly increased in tandem with increasing tertile of serum RCAN2 concentrations (*p* < 0.001).

### Correlations and Regression of Serum RCAN2 Concentrations With Clinical Parameters in the Study Population

Correlation analysis was used to investigate the relationship between metabolic parameters and serum RCAN2 concentrations. As depicted in **Supplementary Table 2**, serum RCAN2 concentrations were positively correlated with age, BMI, BW, SBP, DBP, WC, HC, WHR, ALT, AST, TP, ALB, GLO, GGT, Urea, UA, TC, TG, LDL-C, FBG and height; and negatively correlated with AST/ALT, DBIL, HDL-C (all *p* < 0.05) in all subjects. In the group with normal weight, serum RCAN2 concentrations were positively correlated with BW, height, WHR, SBP, DBP, ALT, AST, TP, ALB, GLO, GGT, TC, TG, LDL-C and FBG (all *p* < 0.05). In the overweight group, serum RCAN2 concentrations were positively correlated with age, BW, WHR, SBP, DBP, ALT, AST, IBIL, GGT, UA, Crea, TC and TG, negatively correlated with AST/ALT and eGFR (all *p* < 0.05). In the group with obesity, serum RCAN2 concentrations were positively correlated with AST/ALT, UA, TC and TG, and negatively correlated with WBC, NEU, and DBIL (all *p* < 0.05).

**Figure 2:**
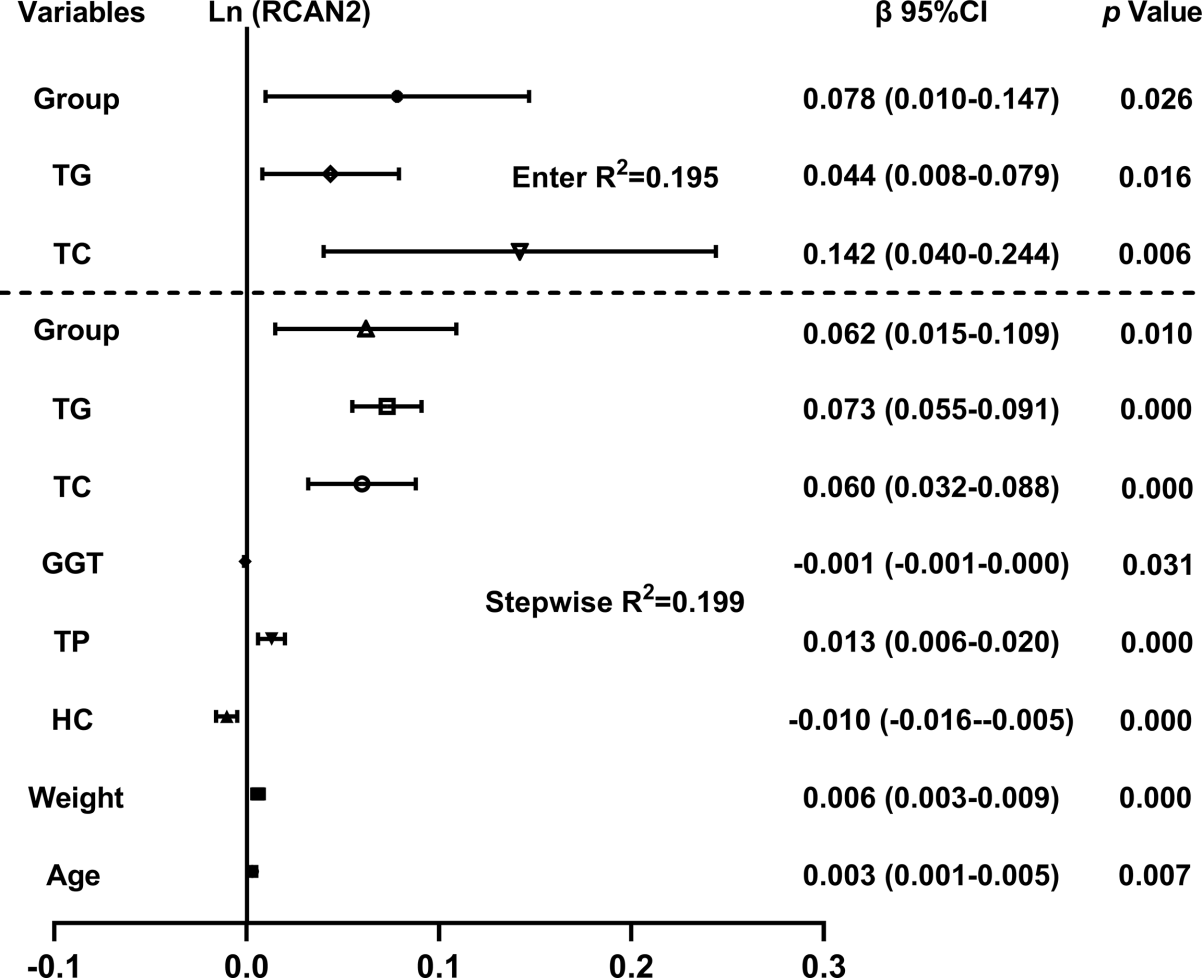
Multiple regression analysis of variables independently related to serum RCAN2 concentrations in all subjects. The regression coefficients (β) and 95% confident interval (CI) from linear regression analysis were displayed. Serum RCAN2 concentrations were ln-transformed before analysis. The statistic of group: 1=NW, 2=OW, 3=OB.

The independent variables associated with the concentration of serum RCAN2 were discussed by multiple linear regression analysis. As shown in **Figure 2**, all factors enter and stepwise regression analyses showed that group, TG and TC were independent variables affecting serum RCAN2 concentrations.

### Association of Serum RCAN2 Concentrations With Overweight and Obesity Risks

To investigate the relationship between serum RCAN2 concentrations and the risks of overweight and obesity, participants with overweight/obesity and normal weight were stratified into three parts based on serum RCAN2 tertiles. As shown in **Table 2**, univariate unconditional logistic regression analysis showed that subjects with the highest tertile of RCAN2 concentrations had 1.539-fold higher risk of obesity than those with the lowest tertile of RCAN2 concentrations (OR = 2.539, 95% CI 1.546-4.170, *p* < 0.001). After adjusting for age and sex in Model 1, the tendency still existed with an OR of 2.509 (95% CI 1.521-4.138, *p* < 0.001). In model 2, even further adjusting for WC, HC, WHR, WBC, NEU, ALT, AST, AST/ALT, TP, ALB, GLO, A/G, TBIL, DBIL, GGT, ALP, Urea, UA, Crea, FBG, HCY, eGFR, Height, and heart rate, the increased OR of obesity in the highest RCAN2 concentrations was also observed and higher than in model 1 (OR = 11.496, 95% CI 1.494 -88.475, *p* = 0.019). Similarly, subjects in the highest tertile of RCAN2 concentrations had an increased risk of overweight when compared with those in the lowest tertile of RCAN2 concentrations (Univariate, OR = 1.606, 95% CI 1.123-2.296, *p* = 0.009; Model 1, OR = 1.517, 95% CI 1.054-2.184, *p* = 0.025; Model 2, OR = 1.687, 95% CI 1.006-2.829, *p* = 0.048).

**Table 2:** Unconditional logistic regression analysis of overweight and obesity risks according to the tertiles of serum RCAN2 concentrations and serum RCAN2 concentrations (ng/mL).

Measurement	RCAN2 tertiles			RCAN2 Concentrations (ng/mL)
	Lowest OR (95%CI)	Median OR (95%CI)	Highest OR (95%CI)	
**Range (ng/mL)**	<7.86	7.86-10.63	>10.63	–
**RCAN2 in OW vs. controls**				
**OW/controls**	123/139	139/114	135/95	397/348
**Univariate**	1 (reference)	1.378 (0.974-1.949)	**1.606 (1.123-2.296)**	**1.099 (1.051-1.150)**
***P*-value**		0.070	**0.009**	**<0.001**
**Model 1**	1 (reference)	1.340 (0.945-1.900)	**1.517 (1.054-2.184)**	**1.093 (1.044-1.144)**
***P*-value**		0.101	**0.025**	**<0.001**
**Model 2**	1 (reference)	1.401 (0.873-2.248)	**1.687 (1.006-2.829)**	**1.102 (1.031-1.177)**
***P*-value**		0.163	**0.048**	**0.004**
**RCAN2 in OB vs. controls**				
**OB/controls**	34/139	34/114	59/95	127/348
**Univariate**	1 (reference)	1.219 (0.713-2.084)	**2.539 (1.546-4.170)**	**1.175 (1.112-1.242)**
***P*-value**		0.468	**<0.001**	**<0.001**
**Model 1**	1 (reference)	1.197 (0.697-2.057)	**2.509 (1.521-4.138)**	**1.176 (1.112-1.243)**
***P*-value**		0.515	**<0.001**	**<0.001**
**Model 2**	1 (reference)	0.795 (0.096-6.576)	**11.496 (1.494-88.475)**	**3.244 (1.199-8.777)**
***P*-value**		0.832	**0.019**	**0.020**

Multivariate odds ratios (ORs) and 95% confidence intervals (CIs) from unconditional logistic regression models were applied in the analysis. Bold font indicated p < 0.05.

Model 1: adjusted for age and sex.

Model 2: adjusted for Model 1+ WC, HC, WHR, WBC, NEU, ALT, AST, AST/ALT, TP, ALB, GLO, A/G, TBIL, DBIL, IBIL, GGT, ALP, Urea, UA, Crea, FBG, HCY, eGFR, Height, Heart Rate.

When considered as continuous variables, every 1-unit increase in serum RCAN2 concentrations was associated with 9.9%, 9.3%, and 10.2% increases, respectively, in risk of overweight in univariate model, model 1 and model 2. For obesity risk, in univariate model, model 1 and model 2, every 1-unit increase in serum RCAN2 concentrations was associated with an increased risk of 17.5%, 17.6% and 224.4%, respectively (**Table 2**).

### Diagnostic Value of Serum RCAN2 and RCAN2/(AST/ALT) Concentrations for Obesity

**Figure 3:**
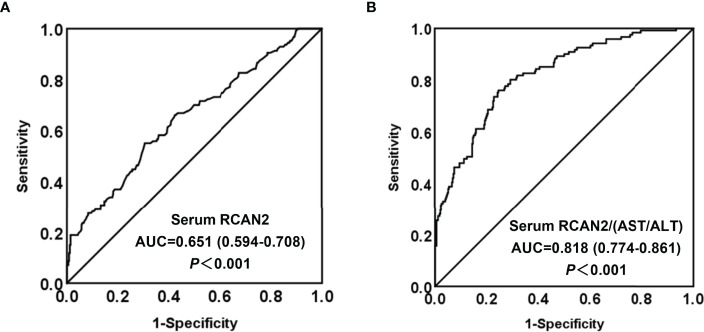
Comparison for ROC curve analysis of serum RCAN2 **(A)**, serum RCAN2/(AST/ALT) **(B)** in participants with OB and NW. ROC curves were derived by plotting the relationship between the specificity and the sensitivity at various cut off concentrations. ROC, receiver-operating characteristic; AUC, area under the curve.

In order to explore the diagnostic value of serum RCAN2 concentrations for obesity, ROC curve analysis was conducted. The ROC displayed in **Figure 3A** represent the diagnostic value of serum RCAN2 for distinguishing the participants with obesity from controls (AUC = 0.651, 95% CI 0.594-0.708, *p* < 0.001, sensitivity = 55.1%, specificity = 69.5%). Diagnosis value accuracy was improved when use serum RCAN2/(AST/ALT) ratio as a biomarker, with AUC of 0.818 (95% CI 0.774-0.861, *p* < 0.001), sensitivity of 76.0% and specificity of 75.4% (**Figure 3B**).

## Discussion

RCAN2 has been reported to significantly increase body weight in animal studies (17). To our knowledge, there is currently no evidence that serum RCAN2 concentrations are associated with overweight/obesity in humans. This study was the first clinical study to reveal the relationship between serum RCAN2 concentrations and overweight/obesity. In this present cross-sectional study, we found that serum RCAN2 concentrations were significantly increased in participants with overweight/obesity compared to participants with normal weight. Additionally, binary logistic regression analysis showed that serum RCAN2 concentration was associated with overweight/obesity. Finally, serum RCAN2, especially RCAN2/(AST/ALT) ratio was a candidate biomarker for the diagnostic of overweight/obesity.

In this study, serum RCAN2 concentrations were significantly correlated with BW, BMI, WHR, SBP, DBP, TC, TG, HDL-C and LDL-C. As we all know, these are adiposity-associated characteristics (20, 21). Overweight, obesity and weight gain are associated with increased risk of CVD like hypertension, and a significantly increased risk of mortality compared to people with normal BMI (22, 23). Fat accumulation, especially visceral adipose tissue accumulation leading to immune cell infiltration and increased secretion of vasoconstrictor mediators is an important cause of obesity promoting hypertension (24). The calcineurin inhibitors (CNIs; cyclosporine A and tacrolimus), whose use have been connected with several cardiovascular side effects such as hypertension, reduce eNOS activity and vasodilation after acetylcholine (ACh) stimulation (25, 26). We hypothesize that RCAN2 may affect blood pressure by indirectly influencing lipid metabolism leading to vascular lipid deposition and directly affecting vascular diastolic function.

Recently, Sun et al. found that knockout of RCAN2 in whole organism in mice can significantly reduce the age- and high-fat diet-induced obesity when compared to the wild type mice (17). Our results also showed that serum RCAN2 concentrations were significantly positively associated with age and body weight in all subjects. Furthermore, findings of the study imply that men and women have different RCAN2 levels. Previous research has demonstrated that the ability of RCAN2 to promote weight gain is diminished by 17β-estradiol-mediated energy depletion in female mice (27), and we speculate that perhaps this hormone may affect RCAN2 levels and antagonize each other with RCAN2 in affecting body weight.

Participants with the highest serum RCAN2 tertile had a significantly higher prevalence of overweight/obesity than subjects with the lowest serum RCAN2 tertile. A considerable percentage of persons with obesity do not have metabolic abnormalities, whereas people with normal body weight acquire metabolic disorders, this unusual phenomenon may be related to visceral fat deposition (28, 29). Abnormal lipid deposition may be the cause of medium/high RCAN2 in some subjects with normal weight.

Energy homeostasis is maintained through complex and interrelated regulatory processes, including the control of appetite and/or satiety mediated by neuroendocrine signals (30). RCAN2 has been reported to be an inhibitor of calcium phosphatase (9, 10); however, its distribution rarely coincides with that of calcineurin in most brain areas (31), and there was no significant difference in hypothalamic calcium phosphatase activity between RCAN2^-/-^ and wild type mice (17). Therefore, RCAN2 may have a function independent of the inhibition of calcineurin (31). Hypothalamus communicates hunger and satiety, regulates appetite and other physiological functions, thereby participating in energy balance (32, 33). RCAN2 was widely expressed in the brain, particularly in dorsomedial (DMH), ventromedial (VMH), paraventricular (PVH) and other hypothalamic nuclei (17). These specialized hypothalamic nuclei sense and integrate signals of diverse hormones and nutrients, changing the expression, secretion and activity of specific neurotransmitters and neuromodulators (34). Therefore, we believed that RCAN2 may be involved in energy homeostasis mechanisms and its function on body weight is independent of leptin (17).

RCAN2 has two primary transcripts, RCAN2-3 and RCAN2-1 (formerly named ZAKI-4α and ZAKI-4β) (31, 35). RCAN2-3 is mainly distributed in the brain, while RCAN2-1 is mainly distributed in brain, heart, skeletal muscles, kidney, and liver (10). mRNA expression of RCAN2-3 was reduced in the cerebral cortex of hypothyroid mice. Injection of L-T_3_ resulted in increased expression of RCAN2-1 mRNA in the heart, whereas the expression of RCAN2-3 and RCAN2-1 mRNA was affected by thyroid hormone status in liver (10). In all subjects tested for FT_3_, there was no statistical difference in circulating FT_3_ concentrations between groups with normal weight, overweight and obesity (**Supplementary Figure 1A**). Meanwhile, subjects were divided into three groups according to the tertiles of serum RCAN2 concentrations. There were also no significant differences in the concentrations of circulating FT_3_ concentration among the three groups (**Supplementary Figure 1B**). Correlation analysis also indicated that serum RCAN2 concentrations were not associated with FT_3_ (data not shown). Hence, circulating FT_3_ may not affect serum RCAN2 concentrations.

The present study also has several limitations worth mentioning. Firstly, RCAN2 has two different splicing variants: RCAN2-1 and RCAN2-3. RCAN2-3 seems to play a major role in regulating body weight. The relationship between RCAN2-1 and RCAN2-3 and overweight/obesity was not investigated separately, so it is not clear which splicing variant is more important in regulating human weight. Secondly, all participants in this study were primarily from China. Therefore, the extrapolation of serum RCAN2 in participants with overweight/obesity diagnosed by BMI criteria should be conducted in other races. Thirdly, this is a cross-sectional study and therefore no causal a conclusion could be drawn between serum RCAN2 concentrations and the increased risk of overweight/obesity. Thus, larger longitudinal intervention studies are needed to confirm our findings in the future. Fourthly, small number and the relatively large span of BMI of participants with obesity led to increased data heterogeneity. In the future research, a larger population and further stratified analysis for obese adults are needed. Finally, all subjects were recruited from physical examination center, which may exist a selection bias. Despite these limitations, this study also has strength. This is the first study to investigate serum RCAN2 concentrations in participants with overweight/obesity and the findings indicated that RCAN2 is a risk factor for overweight/obesity.

## Conclusion

This study found that serum RCAN2 concentrations were significantly increased in patients with overweight and obesity. The percentages of OW/OB subjects gradually increased in tandem with increasing tertiles of RCAN2. The increased serum RCAN2 concentrations were associated with the increased risks of overweight/obesity. Serum RCAN2, especially RCAN2/(AST/ALT) ratio might be the candidate diagnostic markers for obesity.

## Data Availability Statement

The original contributions presented in the study are included in the article/**Supplementary Material**. Further inquiries can be directed to the corresponding authors.

## Ethics Statement

The studies involving human participants were reviewed and approved by Human Research Ethics Committee of the Southwest Medical University Hospital (license number: KY2021086). The patients/participants provided their written informed consent to participate in this study.

## Author Contributions

JF and YX conceived, designed and supervised the study; XT, QR, YZ, and TY provided research guidance; HW, XF, and QR collected the data and biological samples; HW, XF, and QR performed the measurements of serum RCAN2 concentrations; HW and XF analyzed the data and wrote the manuscript; and QR, YZ, XT, TY, JF, and YX critically reviewed and edited the manuscript. All authors read and approved the final manuscript.

## Funding

The Natural Science Foundation of China (grant no. 81970676), the Doctor Initiation Fund of the Affiliated Hospital of Southwest Medical University (grant no. 19064), the Key Project of Natural Science of Southwest Medical University (grant no. 2020ZRZD003).

## Conflict of Interest

The authors declare that the research was conducted in the absence of any commercial or financial relationships that could be construed as a potential conflict of interest.

## Publisher’s Note

All claims expressed in this article are solely those of the authors and do not necessarily represent those of their affiliated organizations, or those of the publisher, the editors and the reviewers. Any product that may be evaluated in this article, or claim that may be made by its manufacturer, is not guaranteed or endorsed by the publisher.
