# Supplementary material for: Association Between Circulating Regulator of Calcineurin 2 Concentrations With Overweight and Obesity
Source: Front Endocrinol (Lausanne). 2022 Jun 6;13:857841. doi: 10.3389/fendo.2022.857841 (PMC9208054; doi:10.3389/fendo.2022.857841)
Supplement: Supplementary file 1 [file DataSheet_1.docx]

**Supplementary Table 1** Comparison of parameters of study population by serum RCAN2 concentrations.

| Variables | Lowest RCAN2  (*n*=296) | Median RCAN2  (*n*=287) | Highest RCAN2  (*n*=289) | *P* value |
| --- | --- | --- | --- | --- |
| Overweight | 123(41.6) | 139 (48.4) | 135 (46.7) |  |
| Obesity | 34 (11.5) | 34 (11.9) | 59 (20.4) |  |
| Anthropometric parameters | | | | |
| Male | 170 (57.4) | 185 (64.5) | 203 (70.2) |  |
| Age (year) | 37.95 ± 10.21 | 40.05 ± 10.93 | 40.79 ± 10.63^a^ | **0.003** |
| BW (kg) | 66.68 ± 13.06 | 66.95 ± 10.34 | 70.67 ± 13.11^ab^ | **<0.001** |
| Height (cm) | 164.42 ± 7.61 | 164.46 ± 7.93 | 165.70 ± 8.17 | **0.045** |
| BMI (kg/m^2^) | 24.56 ± 3.55 | 24.70 ± 3.04 | 25.67 ± 3.83^ab^ | **<0.001** |
| WC (cm) | 82.96 ± 9.14 | 83.53 ± 9.06 | 85.40 ± 9.55^a^ | **0.005** |
| HC (cm) | 97.36 ± 6.52 | 96.42 ± 6.40 | 97.52 ± 6.96 | 0.103 |
| WHR | 0.85 ± 0.06 | 0.87 ± 0.06^a^ | 0.87 ± 0.05^a^ | **<0.001** |
| SBP (mmHg) | 119.05 ± 13.60 | 121.67 ± 13.14^a^ | 125.24 ± 15.70^ab^ | **<0.001** |
| DBP (mmHg) | 71.40 ± 10.47 | 73.65 ± 9.30^a^ | 75.14 ± 10.74^a^ | **<0.001** |
| Heart Rate | 82.89 ± 11.25 | 84.90 ± 11.15 | 83.83 ± 11.19 | 0.052 |
| Metabolic parameters | | | | |
| WBC (10^9/L) | 6.34 ± 1.69 | 6.34 ± 1.57 | 6.42 ± 1.50 | 0.349 |
| NEU (10^9/L) | 3.76 ± 1.28 | 3.70 ± 1.26 | 3.65 ± 1.11 | 0.797 |
| ALT (U/L) | 25.87 ± 20.57 | 28.77 ± 19.26^a^ | 33.13 ± 30.17^a^ | **<0.001** |
| AST (U/L) | 22.19 ± 8.07 | 23.71 ± 8.53^a^ | 25.91 ± 13.19^a^ | **<0.001** |
| AST/ALT | 1.05 ± 0.42 | 0.97 ± 0.37^a^ | 0.96 ± 0.36^a^ | **0.007** |
| TP (g/L) | 71.86 ± 3.14 | 72.40 ± 3.29 | 72.87 ± 3.52^a^ | **0.004** |
| ALB (g/L) | 46.32 ± 2.21 | 46.51 ± 2.32 | 46.83 ± 2.33^a^ | **0.039** |
| GLO (g/L) | 25.55 ± 2.50 | 25.89 ± 2.72 | 26.04 ± 2.81 | 0.193 |
| A/G | 1.83 ± 0.21 | 1.82 ± 0.23 | 1.82 ± 0.22 | 0.486 |
| TBIL (μmol/L) | 15.41 ± 6.27 | 15.34 ± 6.69 | 15.24 ± 6.09 | 0.916 |
| DBIL (μmol/L) | 4.53 ± 1.84 | 4.33 ± 1.86 | 4.08 ± 1.72^a^ | **0.003** |
| IBIL (μmol/L) | 10.89 ± 4.60 | 11.01 ± 4.98 | 11.16 ± 4.54 | 0.590 |
| GGT (U/L) | 27.71 ± 29.30 | 35.55 ± 41.23^a^ | 39.17 ± 36.80^a^ | **<0.001** |
| ALP (U/L) | 71.53 ± 19.76 | 72.69 ± 20.56 | 74.15 ± 19.40 | 0.253 |
| Urea (mmol/L) | 4.92 ± 1.16 | 5.00 ± 1.12 | 5.07 ± 1.01 | 0.167 |
| UA (μmol/L) | 332.49 ± 81.23 | 347.85 ± 78.20 | 360.78 ± 91.72^a^ | **0.001** |
| Crea (μmol/L) | 65.13 ± 12.19 | 67.44 ± 12.63 | 67.15 ± 12.17 | 0.051 |
| TC (mmol/L) | 4.54 ± 0.77 | 4.81 ± 0.86^a^ | 5.12 ± 0.90^ab^ | **<0.001** |
| TG (mmol/L) | 1.31 ± 0.79 | 1.56 ± 0.80^a^ | 2.31 ± 2.37^ab^ | **<0.001** |
| HDL-C (mmol/L) | 1.35 ± 0.32 | 1.31 ± 0.34 | 1.28 ± 0.34^a^ | **0.012** |
| LDL-C (mmol/L) | 2.98 ± 0.76 | 3.24 ± 0.84^a^ | 3.34 ± 0.99^a^ | **<0.001** |
| FBG (mmol/L) | 5.22 ± 1.30 | 5.14 ± 0.73 | 5.42 ± 1.60^a^ | **0.028** |
| HCY (μmol/L) | 11.75 ± 6.63 | 12.04 ± 6.48 | 12.66 ± 8.81 | 0.242 |
| eGFR（ml/min） | 125.67 ± 22.04 | 121.22 ± 20.55^a^ | 123.00 ± 21.24 | **0.033** |
| RCAN2 (ng/mL) | 6.36 ± 1.05 | 9.16 ± 0.79^a^ | 14.09 ± 3.96^ab^ | **<0.001** |

Continuous variables were expressed as mean ± SD. Categorial variables were expressed as *n* (%).

*P* values were derived from Student’s *t* test or the Mann–Whitney *U* test and one-way analysis of variance (ANOVA) or Kruskal-Wallis test for continuous variables according to the data distribution. ^a^*P* < 0.05 compared with lowest RCAN2 group; ^b^*P* < 0.05 compared with median RCAN2 group. Bold font indicated *P* < 0.05.

**Supplementary Table 2** Bivariate correlation between serum RCAN2 concentrations and other parameters.

| Variables | Serum RCAN2 concentrations (ng/mL) | | | | |
| --- | --- | --- | --- | --- | --- |
|  | **Total** | **Normal Weight** | **Overweight** | **Obesity** | |
| Anthropometric parameters | | | | | |
| Sex | **-0.103** | -0.105 | **-0.142** | -0.046 | |
| Age (year) | **0.087** | 0.105 | **0.112** | 0.005 | |
| BW (kg) | **0.218** | **0.113** | **0.102** | 0.034 | |
| Height (cm) | **0.097** | **0.122** | 0.080 | 0.090 | |
| BMI (kg/m^2^) | **0.214** | 0.017 | 0.074 | -0.038 | |
| WC (cm) | **0.182** | 0.072 | 0.093 | -0.090 | |
| HC (cm) | **0.100** | -0.065 | -0.078 | -0.071 | |
| WHR | **0.195** | **0.150** | **0.172** | -0.078 | |
| SBP (mmHg) | **0.200** | **0.190** | **0.201** | -0.008 | |
| DBP (mmHg) | **0.181** | **0.149** | **0.199** | -0.021 | |
| Heart Rate | 0.017 | 0.006 | 0.067 | -0.109 | |
| Metabolic parameters | | | | |  |
| WBC (10^9/L) | 0.043 | 0.024 | 0.058 | **-0.184** | |
| NEU (10^9/L) | -0.017 | -0.026 | -0.026 | **-0.195** | |
| ALT (U/L) | **0.121** | **0.106** | **0.164** | -0.086 | |
| AST (U/L) | **0.132** | **0.177** | **0.148** | -0.038 | |
| AST/ALT | **-0.098** | -0.034 | **-0.125** | **0.226** | |
| TP (g/L) | **0.135** | **0.223** | 0.066 | 0.124 | |
| ALB (g/L) | **0.068** | **0.153** | 0.090 | -0.012 | |
| GLO (g/L) | **0.109** | **0.157** | 0.005 | 0.152 | |
| A/G | -0.058 | -0.069 | 0.035 | -0.121 | |
| TBIL (μmol/L) | -0.028 | -0.010 | 0.073 | -0.132 | |
| DBIL (μmol/L) | **-0.119** | -0.094 | -0.027 | **-0.213** | |
| IBIL (μmol/L) | 0.007 | 0.025 | **0.108** | -0.101 | |
| GGT (U/L) | **0.145** | **0.126** | **0.140** | 0.016 | |
| ALP (U/L) | 0.066 | 0.070 | 0.019 | 0.010 | |
| Urea (mmol/L) | **0.070** | 0.086 | 0.051 | 0.135 | |
| UA (μmol/L) | **0.218** | 0.091 | **0.190** | **0.245** | |
| Crea (μmol/L) | 0.053 | 0.055 | **0.114** | -0.026 | |
| TC (mmol/L) | **0.264** | **0.278** | **0.243** | **0.246** | |
| TG (mmol/L) | **0.406** | **0.398** | **0.341** | **0.382** | |
| HDL-C (mmol/L) | **-0.143** | -0.036 | -0.094 | -0.116 | |
| LDL-C (mmol/L) | **0.106** | **0.141** | 0.095 | -0.048 | |
| FBG (mmol/L) | **0.097** | **0.237** | 0.080 | -0.129 | |
| HCY (μmol/L) | 0.003 | 0.079 | 0.030 | -0.152 | |
| eGFR（ml/min） | -0.032 | -0.040 | **-0.103** | 0.063 | |

Pearson’s correlation tests were used. Bold font indicated *P* < 0.05.

*
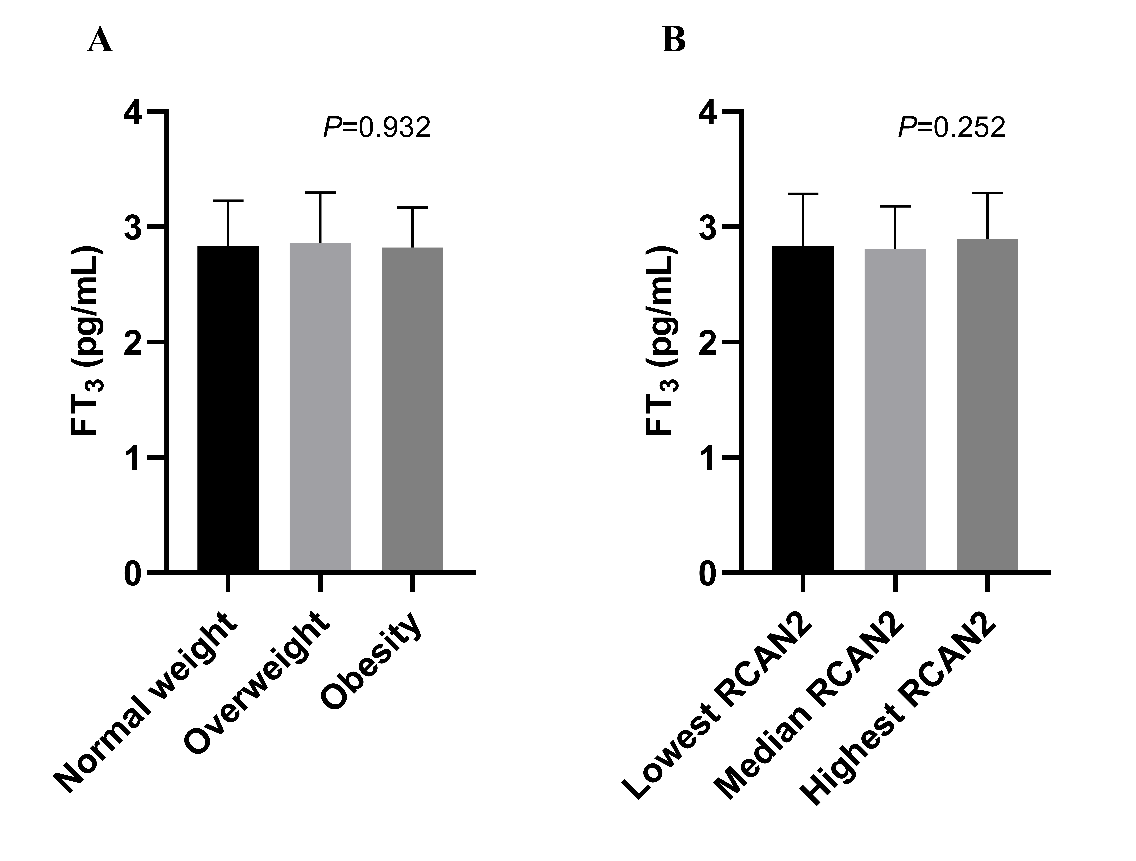
*

**Supplementary Figure 1** Serum FT_3_ concentrations in participants with NW, OW and OB (A). Serum FT_3_ concentrations in lowest, median and highest RCAN2 (B). FT_3_, free triiodothyronine. *P* values were derived from Kruskal-Wallis test.
